# Pediatric Pan-Central Nervous System Tumor Methylome Analyses Reveal Immune-Related LncRNAs

**DOI:** 10.3389/fimmu.2022.853904

**Published:** 2022-05-04

**Authors:** Yongsheng Li, Sicong Xu, Dahua Xu, Tao Pan, Jing Guo, Shuo Gu, Qiuyu Lin, Xia Li, Kongning Li, Wei Xiang

**Affiliations:** ^1^ College of Biomedical Information and Engineering, NHC Key Laboratory of Control of Tropical Diseases, Hainan Women and Children’s Medical Center, Hainan Medical University, Haikou, China; ^2^ College of Bioinformatics Science and Technology, Harbin Medical University, Harbin, China

**Keywords:** pediatric tumors, immune pathways, cancer subtypes, long non-coding RNAs, DNA methylation

## Abstract

Pediatric central nervous system (CNS) tumors are the second most common cancer diagnosis among children. Long noncoding RNAs (lncRNAs) emerge as critical regulators of gene expression, and they play fundamental roles in immune regulation. However, knowledge on epigenetic changes in lncRNAs in diverse types of pediatric CNS tumors is lacking. Here, we integrated the DNA methylation profiles of 2,257 pediatric CNS tumors across 61 subtypes with lncRNA annotations and presented the epigenetically regulated landscape of lncRNAs. We revealed the prevalent lncRNA methylation heterogeneity across pediatric pan-CNS tumors. Based on lncRNA methylation profiles, we refined 14 lncRNA methylation clusters with distinct immune microenvironment patterns. Moreover, we found that lncRNA methylations were significantly correlated with immune cell infiltrations in diverse tumor subtypes. Immune-related lncRNAs were further identified by investigating their correlation with immune cell infiltrations and potentially regulated target genes. LncRNA with methylation perturbations potentially regulate the genes in immune-related pathways. We finally identified several candidate immune-related lncRNA biomarkers (i.e., SSTR5-AS1, CNTN4-AS1, and OSTM1-AS1) in pediatric cancer for further functional validation. In summary, our study represents a comprehensive repertoire of epigenetically regulated immune-related lncRNAs in pediatric pan-CNS tumors, and will facilitate the development of immunotherapeutic targets.

## Introduction

Pediatric central nervous system (CNS) tumors are the second most common cancer among children (1), which form a heterogeneous group of tumors and are responsible for the highest number of cancer-related deaths in children (2). Genomic studies have revealed a number of genomic variants across new diagnoses as well as relapsed pediatric cancers (3, 4). It is now well established that high-throughput next-generation sequencing (NGS) approaches add significant value for cancer diagnoses and prognostic and therapeutic targets (5, 6). However, the discovery of pediatric cancer-associated alterations has primarily focused on genetic variants. Epigenome alterations that contribute to deregulate transcription and promote oncogenic pathways in pediatric cancer are still largely unknown.

DNA methylation has a substantial impact on gene expression, affecting the development and progression of cancer (7). Emerging NGS technologies have increased our understanding of the DNA methylation in pediatric cancer biomarker identification, subtype classification, and prognostication (8). For example, the methylation age has been found to have potential as a prognostic biomarker in pediatric tumors (9). Genome-wide DNA methylation studies have identified hyper-methylated genes involved in tissue development in pediatric embryonic and alveolar rhabdomyosarcomas (10). DNA methylation profiles have been widely used to sub-classify CNS tumors, including pediatric cancer (11). These data highlight the important roles of DNA methylation in diverse pediatric CNS tumors. However, the majority of these studies focused on the DNA methylation perturbation in protein-coding genes, particularly the promoter regions.

Deep sequencing with new computational approaches for assembling transcriptome has identified tens of thousands of long noncoding RNAs (lncRNAs) across different tissues and cell types (12, 13). LncRNAs have been found to play important roles in diverse pediatric cancers. HOXD-AS1 was found to control the expression levels of clinically significant protein-coding genes involved in angiogenesis and inflammation in neuroblastomas (14). Linc-NeD125 can function independently of the hosted microRNA, by reducing cell proliferation and activating BCL-2 in neuroblastomas (15). Moreover, DNA methylation has been found to be a fundamental feature of epigenomes that can affect the expression of lncRNAs (16). Although several lncRNA methylation perturbations have been revealed in pediatric cancer, we still lack knowledge on the epigenetic landscapes of lncRNAs in diverse pediatric CNS tumors.

Moreover, immune therapy is an attractive alternative approach for targeting CNS tumors. Several studies have revealed the complex tumor immune microenvironment (TIME) in CNS tumors. For instance, tumor-associated macrophages make up a large proportion of immune cells in glioma patients and are associated with tumor grade (17). Activation of CD8 T cells can establish a neuron–immune–cancer axis, which was responsible for glioma growth (18). A recent study described the TIME of pediatric CNS tumors and identified tumor-specific immune clusters with phenotypic characteristics relevant to immunotherapy response (19). Furthermore, lncRNAs have been demonstrated to play important roles in cancer immunology (20–22). However, only a few immune-related lncRNAs have been identified in pediatric CNS tumors. Therefore, further studies on lncRNAs and their roles in immune regulation will be essential to identify immunotherapy targets in pediatric cancer.

To systematically analyze the epigenetically regulated lncRNAs across pediatric CNS tumors, we integrated the DNA methylation profiles of 2,257 pediatric CNS tumors across 61 subtypes. We revealed prevalent lncRNA methylation and further classified pediatric cancer into 14 lncRNA methylation clusters, which were characterized by distinct phenotypes. We also found that lncRNA methylations are significantly correlated with immune cell infiltrations and potentially regulate immune-related pathways. We show that analysis of lncRNA methylation can identify immune-related lncRNA biomarkers in pediatric cancers.

## Materials and Methods

### DNA Methylation Across Pediatric Tumors

DNA methylation profiles across pediatric CNS tumors were collected from Gene Expression Omnibus (GEO) and ArrayExpress (**Table S1**). Here, an Illumina HumanMethylation450 BeadChip was used for profiling the DNA methylation in pediatric cancers (19). In total, genome-wide DNA methylation profiles of 2,257 pediatric CNS tumors (age < 19 years old) across 61 World Health Organization (WHO) histopathological entities were extracted. To include the comprehensive pediatric cancer samples, we included all patients in our analysis. The raw “.idat” files were background-corrected, single-sample Noob and functional normalized (23) based on the “preprocessFunnorm” module in the minfi v.1.34.0 package (24).

### LncRNA Annotations and Genomic Features

Genome-wide annotations of lncRNAs were downloaded from GENCODE (V34, GRCh38) (25). The numbers of exons for lncRNAs were calculated based on exon annotation of lncRNAs. The evolutionary conservation scores for lncRNAs were obtained using the phastCons100way.UCSC.hg38 (3.7.1) R package (26). The GC content and normalized CpG fraction (observed CpG/expected CpG) were calculated based on the sequences of lncRNAs, where the expected CpG was calculated as (GC content/2)^2^ (27).

### LncRNA Methylation

The genomic locations of DNA methylation probes were first transferred from GRCh37 to corresponding coordinates in GRCh38 through UCSC LiftOver (https://genome.ucsc.edu/cgi-bin/hgLiftOver). Next, the probes were filtered similar to one of the previous studies (11). First, the probes that were located on chromosomes X and Y were excluded. Next, the probes that included a single-nucleotide polymorphism (SNP) and those not mapping uniquely to the CRCh37 were excluded. In addition, we excluded probes that were not included in the Illumina EPIC array.

Next, we mapped the probes to the promoter regions of lncRNAs and protein-coding genes. Promoter regions were defined as the 4-kb regions centered at the transcriptional start sites (TSSs) of lncRNAs and genes (28, 29). In total, 56,072 and 156,664 probes were mapped to 9,389 and 17,762 protein-coding genes, respectively.

### Classification of LncRNAs

The DNA methylation levels of lncRNAs were calculated as the average beta values of probes that mapped to the promoter regions (30). Next, lncRNAs were classified into three categories: (i) hyper-methylated lncRNAs were defined as those with methylation levels > 0.7 in more than 80% samples; (ii) hypo-methylated lncRNAs were defined as those with methylation levels < 0.3 in more than 80% samples; and (iii) other lncRNAs not in the hyper- or hypo-methylated group were defined as inter-methylated lncRNAs.

The genomic differences (such as GC contents and normalized CpG fractions) among three lncRNAs groups were evaluated by Kolmogorov–Smirnov tests. Differences in conservation scores, number of CpGs, and exons were evaluated by Wilcoxon’s rank sum tests.

### Identification of Tumor Subtypes Based on LncRNA methylation

First, we selected the top 5% probes with high variation (S.D. > 0.267) that mapped to lncRNA promoters. The DNA methylation profiles of variable CpG sites were used for clustering the pediatric CNS tumor samples. The ConsensusClusterPlus package was used to identify the cancer subtypes (31), with 50 iterations and a resample rate of 0.8. The number of clusters ranged from *k* = 2 to 60. In addition, Pearson correlation coefficient was calculated for paired samples based on the DNA methylation profiles.

### Likelihood Ratio Test

The Cox proportional hazards model was first constructed based on clinical features, including gender and age. Next, another two models that added WHO catalogs and lncRNA-based subtypes were used. Clinical clusters represent the Cox model constructed by gender and age. Original clusters represent the Cox model constructed by gender, age, and WHO category. LncRNA clusters represent the Cox model constructed by gender, age, WHO category, and lncRNA clusters. We estimated the likelihood ratio (LR) statistic of three regression models and the changes in LR were assessed by Chi-square test (32).

### Immune Cell Infiltration in Pediatric Tumors

To estimate the immune cell infiltration in pediatric tumors, we first downloaded the signature matrix “StromalMatrix_V2” from MethyCIBERSORT (33). CpG signatures were provided for CD14+ (monocyte lineage), CD19+ (B-lymphocytes), effector lymphocytes (CD4_Eff), CD56+ (NK cells), CD8 (cytotoxic T-lymphocytes), endothelial cells, eosinophils (Eos), fibroblasts, neutrophils (Neu), and regulatory T lymphocytes (Treg). Next, the DNA methylation profiles were subjected into CIBERSORT with the DNA methylation signature (34). CIBERSORT was run based on 100 permutations without quantile normalization.

### Immune Signatures

The immune signatures gene sets, including immune checkpoints, immune cytolytic activity (CYT), human leukocyte antigen (HLA), interferon (IFN) response, and tumor-infiltrating lymphocytes (TILs), were obtained from a previous study (35). In addition, 301 CpG probes that could predict the response to anti-PD-1 treatment in non-small cell lung cancer were collected (36). Another 67 immune cell type-specific gene–CpG pairs for 21 immune cell populations were collected from literature (37). The average methylation levels of related genes were used to estimate the DNA methylation activity of immune signatures. All CpG sites related to immune signatures are provided in **Table S2**.

### Identification of Immune Cell Infiltration-Related LncRNAs

To identify immune cell infiltration-related lncRNAs, we calculated the Spearman correlation coefficient (SCC) between immune cell infiltration levels and lncRNA methylation in a specific pediatric CNS tumor subtype. LncRNAs with absolute SCC > 0.3 and *p*-adjusted < 0.05 were considered as immune cell infiltration-related lncRNAs.

### Identification of LncRNAs Associated With Immune-Related Pathways

First, 1,811 protein coding genes involved in 17 immune-related pathways were obtained from one of our previous studies (20). Next, we identified the protein-coding gene within 10 kb for each immune cell infiltration-related lncRNA based on genomic locations. If the protein-coding gene within 10 kb was annotated in immune-related pathways, we considered this lncRNA to be involved in the regulation of immune-related pathways.

### Differential Methylation and Survival Analysis

We compared the DNA methylation differences of lncRNAs based on *t*-test in a “one vs. rest” way. LncRNAs with an absolute value of methylation difference greater than 0.2 and a *p*-value less than 0.05 were considered to be differentially methylated.

Tumor samples were divided into two groups based on the DNA methylation level of lncRNAs. The cutoff was determined by the internal R function “survminer: surv_cutpoint”. The difference in overall survival between two groups was assessed by the log-rank test.

## Results

### Landscape of LncRNA Methylation in CNS Tumors

**Figure 1:**
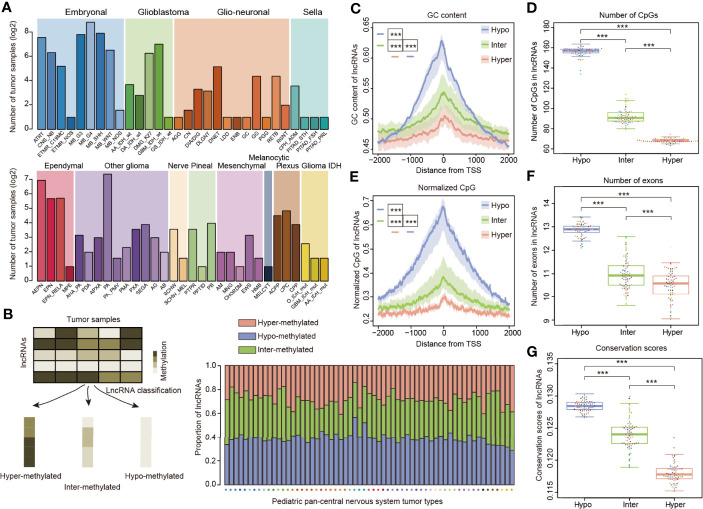
LncRNA methylation patterns in pediatric pan-CNS tumors. **(A)** Number of tumor samples across different types of pediatric CNS tumors. The patients were classified into 12 major clusters with 61 sub-clusters. **(B)** Method to define the lncRNAs with different methylation patterns. The bar plots in the lower third show the proportion of lncRNAs with different methylation patterns across cancer types. **(C)** Distributions of GC content around the TSSs for lncRNAs with different methylation patterns. **(D)** Box plots showing the number of CpGs in lncRNA promoters with different methylation patterns. **(E)** Distributions of normalized CpG around the TSSs for lncRNAs with different methylation patterns. **(F)** Box plots showing the number of exons in lncRNAs for lncRNAs with different methylation patterns. **(G)** Box plots showing the average conservation scores in lncRNA promoters with different methylation patterns. *** indicates p < 0.001.

To investigate the methylation of lncRNAs, we obtained the genome-wide DNA methylation profiles for 2,257 pediatric tumor samples. In order to account for molecular or histological subtypes, these samples were further stratified into a total of 12 main classes, including 61 WHO-defined CNS tumor subtypes (**Figure 1A**), as previously defined (11). Next, we determine lncRNA methylation in cancers and classified lncRNAs into three categories according to the methylation profiles of pediatric tumors (**Figure 1B**). Approximately 30% of lncRNAs exhibited hyper-methylation and 40% of lncRNAs exhibited hypo-methylation across cancer subtypes (**Figure 1B**).

Since genes or lncRNAs with distinct methylation patterns exhibited different genomic characteristics (30, 38), we next compared the genomic and evolutional features of lncRNAs in three categories. We found that the enrichment of GC content and CpGs in all three types of lncRNAs is symmetric and peaks around the core lncRNA promoters (**Figures 1C, D**). In addition, hypo-methylated lncRNAs had significantly higher GC content and number of CpGs than the other two categories (**Figures 1C, D**, *p* < 0.001). As the number of CpGs might be affected by the length or GC content of lncRNAs, we thus calculated the normalized CpGs of lncRNAs. We found that hypo-methylated lncRNAs also had significantly higher normalized CpGs than the other two groups (**Figure 1E**, *p*-values < 0.001). These results were consistent with observations in protein-coding genes that high-CpG-content genes are hypo-methylated (27).

While lncRNAs exons are much less conserved than protein-coding genes (12), it is unknown whether the lncRNAs with different methylation patterns evolve in a distinct way. First, we compared the number of exons in lncRNAs and found that hypo-methylated lncRNAs had more exons than other lncRNAs (**Figure 1F**, *p*-values < 0.001). To quantify the evolutionary conservation of lncRNA promoters, we used phastCons scores of placental mammals (26). We compared the average conservation score of lncRNAs and found that hypo-methylated lncRNAs had significantly higher conservation scores (**Figure 1G**, *p*-values < 0.001). Similarly, we obtained the same results when we analyzed the pediatric cancer individually (**Figure S1**).

### LncRNA Methylation Heterogeneity in CNS Tumors

**Figure 2:**
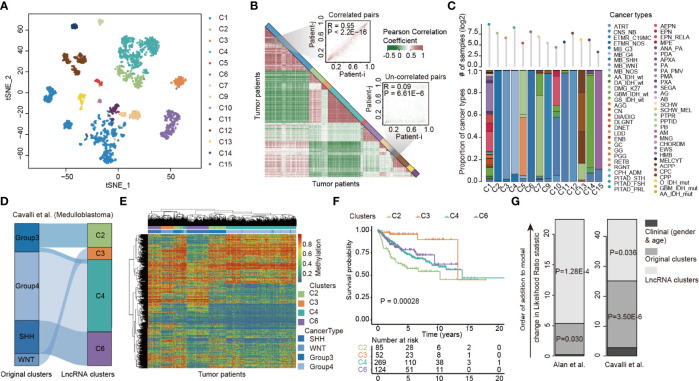
LncRNA methylation heterogeneity in pediatric pan-CNS tumors. **(A)** Unsupervised clustering of tumor samples using tSNE dimensionality reduction. Individual samples are color-coded in the respective class color. **(B)** Heat maps showing the correlations of lncRNA methylation among tumor samples. Two representative examples were shown in the right side. **(C)** Bar plots showing the proportion of cancer types in each lncRNA methylation cluster. **(D)** Alluvial diagram of lncRNA methylation clusters in groups with different molecular subtypes. **(E)** Heat maps showing the methylation of lncRNAs in different clusters. **(F)** Kaplan–Meier curves of overall survival for patients with lncRNA methylation clusters. **(G)** The estimated log-likelihood ratio statistic of a Cox proportional hazards model. The change of LR statistic as features were added to the model was assessed for significance by Chi-square tests. Clinical clusters represent the Cox model constructed by gender and age. Original clusters represent the Cox model constructed by gender, age, and WHO category. LncRNA clusters represent the Cox model constructed by gender, age, WHO category, and lncRNA clusters.

Previous studies have reported substantial variability in the histopathological diagnosis of many CNS tumors (39). We next investigated the extent of the lncRNA methylation heterogeneity in pediatric CNS tumors. Consensus clustering of lncRNAs with variable DNA methylation identified 14 optimal subtypes that we refer to as C1–C7 and C9–C15 (**Figure 2A** and **Figure S2**, **Table S3**). Moreover, we visualized the Pearson correlation coefficient (PCC) of lncRNA methylations among tumor patients and found that patients clustered in the same clusters exhibited higher similarity in lncRNA methylation (**Figure 2B**). We next analyzed the compositions of cancer types in 14 lncRNA methylation clusters. Patients in several lncRNA clusters (i.e., C2, C3, C4, and C12) predominantly had an individual cancer type, while patients in C1, C5, C10 and C13 diverse types of pediatric cancer (**Figure 2C**). In particular, the patients in C1 were much heterogeneous, including 652 patients from 48 cancer types.

We next sought to characterize a more refined lncRNA methylation pattern in a single tumor type. We focused on medulloblastoma, which was classified into four subtypes (Group 3, Group 4, SHH, and WNT) in a previous study (40). We found that although the majority of patients in original clusters were classified into the same lncRNA methylation clusters, different lncRNA methylation characteristics were found in the patients of Group 3 (**Figure 2D**). The heat map corresponding to the lncRNA methylation clusters was generated, and we found that lncRNAs exhibited distinct methylation across clusters (**Figure 2E**).

To investigate whether the difference in lncRNA methylation pattern had clinical implications, we next performed Kaplan–Meier survival analysis. We found that the medulloblastoma patients in different lncRNA methylation clusters had significantly higher survival rates (**Figure 2F**, log-rank *p* = 0.00028). Moreover, we obtained similar results in another cancer type (**Figure S3**). However, it is not yet clear whether lncRNA methylation can provide additional prognostic power beyond original cancer types and clinical features. We thus performed a multivariate Cox proportional hazards analysis (32). In this model, we included age, sex, and original cancer types. As a result, we observed a large increase in the predictive fit by considering the lncRNA methylation (**Figure 2G**, *p* = 1.28E-4 and 0.036, Chi-square test), supporting the clinical value of lncRNA methylation patterns in cancer.

### Immune Microenvironment Patterns of LncRNA CNS Subtypes

**Figure 3:**
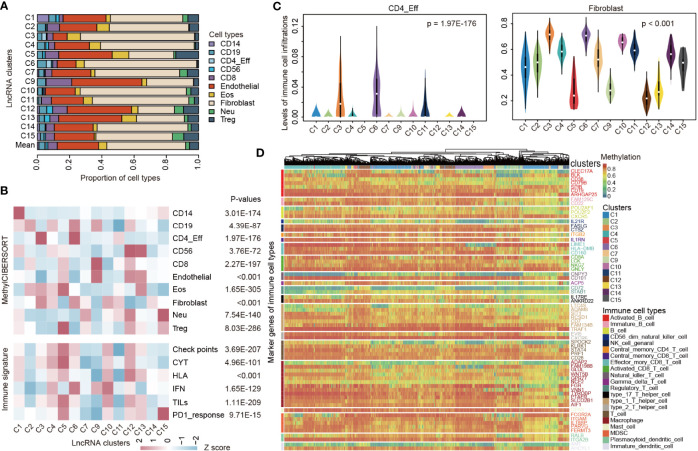
TIME heterogeneity in pediatric pan-CNS tumors. **(A)** The proportions of immune cell-type infiltrations across lncRNA methylation clusters. **(B)** Heat maps showing the average immune cell infiltrations and immune signature scores across lncRNA methylation clusters. *p*-values were for ANOVA tests. **(C)** Violin plots showing the levels of immune cell infiltrations across lncRNA clusters. Left for CD4_Eff cells and right for fibroblast. **(D)** Heat maps showing the relative methylation of immune cell-type marker genes across patients in different lncRNA clusters. Genes were colored based on the immune cell types.

Detailed studies on the TIME are being conducted to uncover the underlying mechanisms of cancer (19). We next estimated the relative proportion of immune cells of patients by methylCIBERSORT (33). In general, we found that there were relatively high proportions of endothelial and fibroblast cells across all pediatric CNS tumors (**Figure 3A**). Moreover, individual lncRNA methylation subtypes varied significantly in the relative proportion of infiltrating cell types (**Figure 3B**). Moreover, we also investigated the immune signature scores and observed high variations among different lncRNA methylation clusters (**Figure 3B**).

For instance, a higher proportion of CD4_Eff was observed in C6 and C3 while limited CD4_Eff was infiltrated in C2 and C4 (**Figure 3C**). No CD4_Eff was infiltrated in C5, C12, and C15 clusters. These observations were consistent with the result that patients in C6 and C3 had better survival than those in C2 and C4 (**Figure 2F**). Although patients in the majority of clusters had a higher infiltration of fibroblast, C5, C9, C12, and C3 patients had a relatively limited infiltration of fibroblast (**Figure 3C**). In contrast, Treg cells were highly infiltrated in patients of C5 (**Figure 3B**) and the methylation of immune-related signatures was also relatively higher in C5. These observations suggested that regulatory T cells might oppose the recovery of nerve injury or psychological stress in CNS, which is consistent with the result of a recent study (41). Regulatory T cells have been demonstrated to counteract neuropathic pain through inhibition of the Th1 response at the site of peripheral nerve injury (42). Increased percentages of regulatory T cells have been associated with inflammatory and neuroendocrine responses to acute psychological stress (43).

We next investigated the DNA methylation of immune-related signatures and found that the marker genes exhibited diverse DNA methylation across clusters (**Figure 3D**). To systematically identify the pathways regulated by DNA methylation, we identified the quantitative differentially methylated regions (QDMRs) (44, 45). Functional enrichment analysis revealed that genes with differentially methylated patterns were significantly enriched in nervous system-related functions (**Figure S4**). For instance, genes in C7 clusters were enriched in “Fc gamma R-mediated phagocytosis” and “TNF signaling pathway”. Genes in C4 were associated with gliogenesis (**Figure S4**). Together, these results suggested the diverse immune microenvironment patterns of lncRNA pediatric CNS subtypes.

### LncRNA Methylation Associated With Immune Cell Infiltration

**Figure 4:**
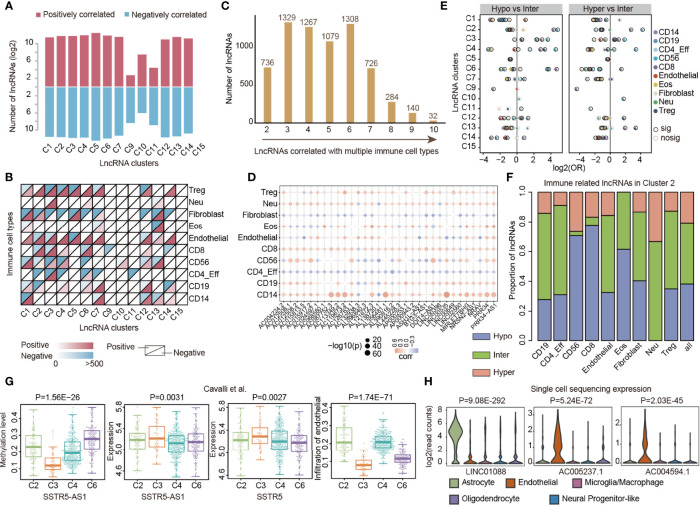
LncRNA methylations correlated with immune cell infiltrations in cancer. **(A)** Bar plots showing the number of lncRNAs in which methylation correlated with immune cell infiltrations across lncRNA clusters. Red for positively correlated lncRNAs and blue for negatively correlated lncRNAs. **(B)** Heat maps showing the number of lncRNAs in which methylation correlated with different types of immune cell infiltrations across lncRNA clusters. **(C)** Number of lncRNAs that correlated with different numbers of immune cell types. **(D)** Balloon plots of correlation between lncRNA methylation and immune cell infiltration. The colors of the balls represent the correlations and sizes represent the –log10(*p*-values). **(E)** Balloon plots showing the enrichment of lncRNAs with different methylation patterns. Balls were colored according to the immune cell types and significant enrichments were colored by dark black margins. **(F)** The proportion of lncRNAs correlated with immune cell infiltrations with different methylation patterns. **(G)** Box plots showing the distribution of methylation, expression, and infiltration of endothelial cells in different lncRNA clusters. **(H)** Violin plots showing the expression of lncRNAs in different immune cells based on single-cell sequencing data.

LncRNAs are emerging as critical regulators of gene expression and they play fundamental roles in immune regulation (20, 28). It is not clear to what extent lncRNA methylations were associated with immune cell infiltration, particularly in pediatric CNS tumors. We thus evaluated the correlation between lncRNA methylation and immune cell infiltrations in each cluster by Spearman correlation coefficient (SCC). In total, we identified 260 to 6,510 lncRNAs, whose methylations were correlated with immune cell infiltration, across 14 clusters (**Figure 4A**). The number of positively correlated lncRNAs was nearly the same as that of the negatively correlated ones. We next analyzed the correlation by immune cell types and found that lncRNA methylation correlated with immune cell infiltration and exhibited a complex pattern across clusters (**Figure 4B**). In general, we identified that lncRNA methylation correlated with diverse immune cell type infiltration in the majority of clusters. However, the majority of lncRNA methylation correlated with CD8 T-cell infiltration in C9 (**Figure 4B**). LncRNA methylation mainly correlated with CD56 and CD4_Eff in C10 and C11 (**Figure 4B**).

In total, we identified 6,901 lncRNAs in which methylation was correlated with at least two types of immune cells (**Figure 4C**). In particular, 32 lncRNAs were correlated with 10 immune cell infiltrations (**Figure 4D**). Interestingly, we found that methylations of these lncRNAs were almost positively correlated with CD8 and CD14 infiltration levels. Next, we further explored the methylation patterns of lncRNAs that were correlated with immune cell infiltrations. We found that hypo-methylated and hyper-methylated lncRNAs were more likely to be correlated with immune cell infiltrations in several clusters when compared with inter-methylated ones (**Figure 4E**), such as in C3 and C4. In particular, we found that hypo-methylated lncRNAs were more likely to be correlated with CD56 and CD8 infiltrations in C2 (**Figure 4F**, *p*-values < 0.05). Moreover, we explored the DNA methylation level of 32 lncRNAs in different clusters. We found that AL121672.3, MIRLET7BHG, PRR34, and PRR34-AS1 were hyper-methylated in C5 (**Figure S5**). LINC00398 was hyper-methylated in C7 and AC012508.1 and AC012508.2 were hyper-methylated in C9. The nearest protein-coding genes of the 32 lncRNAs were enriched in regulation of the muscle system process, negative regulation of the small-molecule metabolic process, and positive regulation of cell death (**Figure S5**).

To investigate the immune regulation of lncRNA methylation, we further analyzed the correlation in medulloblastoma. We identified that SSTR5-AS1 methylation was correlated with endothelial infiltration (**Figure S6**). By integrating DNA methylation and expression profiles, we found that SSTR5-AS1 was hypo-methylated and highly expressed in C3 (**Figure 4G**). Moreover, the potential target gene SSTR5 also exhibited significantly higher expression in patients of C3 (**Figure 4G**, *p* = 0.0027), which had relatively lower endothelial infiltration (**Figure 4G**, *p* = 1.74E-71). These observations suggested that lncRNA methylation might repress the expression of lncRNA first, further decrease the expression of the target gene, and finally repress the immune cell levels. Indeed, it has been demonstrated that SSTR5 can significantly reduce endothelial cell proliferation (46). Moreover, we also identified several lncRNAs that exhibited significantly lower (i.e., LINC01088) or higher (i.e., AC005237.1 and AC004594.1) expressions in endothelial cells based on single-cell data (**Figure 4H**) collected from one recent study (47). Taken together, these results suggest that prevalent lncRNA methylations were associated with immune cell infiltration, which plays important roles in TIME regulation in pediatric CNS tumors.

### Immune-Related LncRNAs Associated With Cancer Subtypes

**Figure 5:**
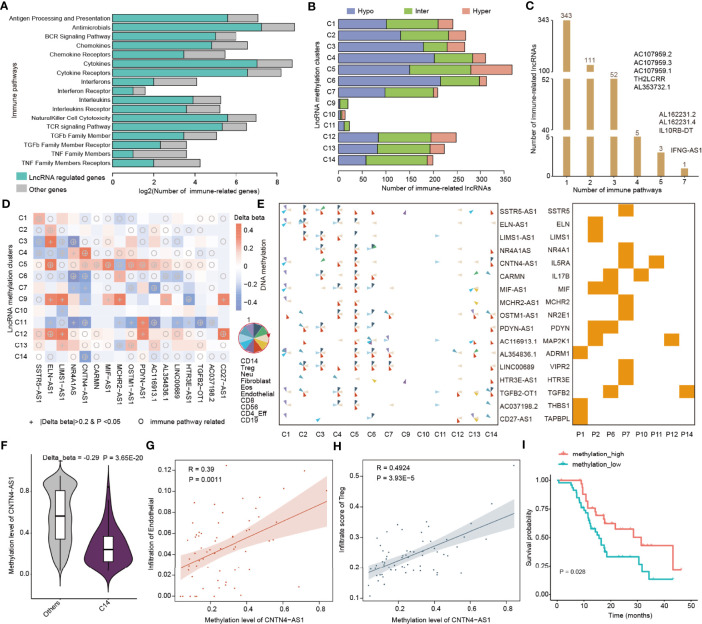
LncRNA methylation involved in immune-related pathways. **(A)** Bar plots showing the number of genes regulated by lncRNAs in each immune-related pathway. The green bars are for lncRNA regulated genes and gray bars are for other genes. **(B)** Bar plots showing the number of immune-related lncRNAs identified in each lncRNA methylation cluster. Red for hyper-methylated lncRNAs, blue for hypo-methylated lncRNAs, and green for inter-methylated lncRNAs. **(C)** Number of lncRNAs that potentially regulate different numbers of immune-related pathways. **(D)** Heat maps showing the differential methylation of lncRNAs across different clusters. The colors represent the difference in DNA methylation levels and “+” indicates that *β* > 0.2 and *p* < 0.05, while the circles indicate that this lncRNA can potentially regulate immune-related pathways. **(E)** Pie charts showing the correlation of lncRNA methylation and immune cell infiltrations in different clusters. The order of the immune cells in the pie is shown in the left side. Right-side heat map showing whether the lncRNA can regulate the corresponding immune-related pathway. **(F)** Violin plots showing the methylation levels of CNTN4-AS1 in C14 and other clusters. **(G, H)** Scatter plots showing the correlation between methylation of CNTN4-AS1 and infiltration of immune cells. **(G)** for endothelial and **(H)** for Treg cells. **(I)** Kaplan–Meier curves of overall survival for patients with high or low CNTN4-AS1 methylation.

To gain insight into the function of lncRNA methylation in immune regulation, we next focused on 17 immunologically relevant gene sets representing distinct immune pathways derived from recent studies (20, 48). We used the protein-coding gene within 10 kb from lncRNAs that were correlated with immune cell infiltrations to predict their functions. We found that the lncRNAs potentially regulate a number of genes in immune-related pathways (**Figure 5A**). In particular, the majority of cytokines and cytokine receptors were regulated by lncRNAs. Next, we analyzed the methylation patterns of immune-related lncRNAs in 14 clusters. We found that there were high numbers of lncRNAs in C5 and C6 clusters, which were mainly hypo-methylated lncRNAs in C6 (**Figure 5B**).

Our previous study has demonstrated that if the lncRNAs can regulate more immune-related pathways, they are more likely to be involved in cancer (20). We next calculated the number of pathways regulated by immune cell infiltration-related lncRNAs. We found that the majority of lncRNAs potentially regulate one or two immune-related pathways and 9 lncRNAs can regulate more than three pathways (**Figure 5C**). In particular, we identified that IFNG-AS1 can regulate 7 immune-related pathways. The lncRNA IFNG-AS1 was found to strongly influence the responses to several pathogens by increasing interferon gamma (IFNγ) secretion (49). IFNG-AS1 is as an important regulator of IFNG expression, which was involved in dynamic and cell state-specific responses to infection (50). These results suggest that the immune-related lncRNAs play important roles in cancer.

Next, we used the “one vs. rest” method to identify the differentially methylated lncRNAs in each cluster. We identified 81 immune-related lncRNAs that were differentially methylated across 14 clusters (**Table S4**). In particular, we found that there were 17 immune-related lncRNAs reported in literature (**Figure 5D**). Moreover, we collected genome-wide DNA methylation from normal controls and compared the methylation level of hub lncRNAs between the CNS tumors and normal tissue. We found that SSTR5-AS1, LIMS1-AS1, MCHR2-AS1, AC116913.1, LINC00689, and TGFB2-OT1 exhibited significantly differential methylation in cancer (**Figure S7**, Wilcoxon’s rank sum test *p*-values < 0.05). The methylations of these lncRNAs were correlated with diverse immune cell infiltrations across 14 clusters, and they can potentially regulate genes in immune-related pathways (**Figure 5E**). We also analyzed the methylation of the genes potentially regulated by lncRNAs and found that the methylation alterations of SSTR5, ELN, MCHR2, PDYN, and TAPBPL were consistent with lncRNAs (**Figure S8**). Next, we investigated to what extent the methylations of lncRNAs were associated with drug treatment response. We first obtained the DNA methylation of lncRNAs of brain tumors from the TCGA cohort. We found that the methylation levels of 12 hub lncRNAs were associated with the drug treatment response (**Figure S9**, Kruskal–Wallis test *p*-values < 0.05). These results suggest that these immune-related lncRNAs might be used for further functional investigation in pediatric CNS tumors.

In particular, we identified the lncRNA CNTN4-AS1, which was involved in neuronal differentiation and gliomagenesis (51). We found that CNTN4-AS1 exhibited significant hypo-methylation in C14 (**Figure 5F** and **Figure S10**, *p* = 3.65E-20), which was mainly formed by IDH wild-type glioma patients. The methylation of CNTN4-AS1 was correlated with endothelial and Treg infiltrations in the C14 cluster (**Figures 5G, H**). We also found that CNTN4-AS1 can regulate IL5RA (**Figure S10**), which plays important roles in the cytokine signaling pathway (52). In addition, we found that high methylation of CNTN4-AS1 was associated with better survival of pediatric glioma patients (**Figure 5I**, log-rank *p* = 0.028). These results suggest that CNTN4-AS1 methylation may be a potential biomarker for pediatric CNS tumor subtypes. We also identified several other biomarkers for pediatric tumor subtypes, such as OSTM1-AS1 and AC116913.1 (**Figures S10–S12**). The methylation level of OSTM-AS1 was correlated with immune cell infiltration in C1, C2, C4–C6, C7, and C12–C14 and altered in C5, C7, and C13 (**Figures S10 and S11**). OSTM-AS1 could potentially target NR2E1 and regulate the cytokine receptor pathway. The methylation level of AC116913.1 was correlated with immune cell infiltration in C2, C4–C7, C11, C13, and C14 and changed in C5, C7, and C11. AC116913.1 could potentially target MP5K1 and regulate the antimicrobials and natural killer cell cytotoxicity pathways (**Figure S8**). All the methylation levels of these lncRNAs were associated with the survival of pediatric gliomas (C7 and C14) (**Figures S10–S12**). We have also explored the DNA methylation level of these three lncRNAs by integrating the GBM and LGG cancer types from TCGA cohorts. We found that these lncRNAs were all hyper-methylated in adult CNS tumors, and the higher methylation level could predict a better overall survival (**Figure S13**).

## Discussion

Epigenetic factors tightly regulate the expression of lncRNAs, which play important roles in cancer development and progression (53). We have demonstrated that lncRNAs exhibited distinct methylation patterns (hyper-, hypo-, and inter-methylated lncRNAs) across pediatric CNS tumors. These lncRNAs had significant genomic features, including GC content, CpG content, number of exons, and conservation. In addition, we have performed lncRNA methylation-based pediatric pan-CNS tumor classification, which will be a valuable asset for clinical decision-making. In particular, we redefined four clusters of medulloblastomas that have different clinical implications. Integrating the lncRNA methylation information can provide additional power for clinical diagnosis.

Moreover, we estimated the TIME of pediatric pan-CNS tumors and found that patients in distinct lncRNA methylation clusters exhibited high heterogeneity in TIME. We demonstrated significant association with lncRNA methylation and immune cell infiltrations across lncRNA clusters. Our results are broadly in accordance with the small number of recent studies on immune infiltration in pediatric CNS tumors (54, 55). We also show that lncRNA clusters are clearly related to the expression of conventional immune targets, such as PDL1, CYT, and IFN. All these studies suggest the important differences in TIME across pediatric brain tumors.

We next explored the associations between lncRNA methylation and immune cell infiltrations. We found that lncRNA methylation was associated with diverse immune cell infiltrations across pediatric pan-CNS tumors. Emerging immune-related lncRNAs are identified in various cancers (22, 56); however, the underlying functions of lncRNAs are still unknown. Promotion or suppression of immune cells may be a major way for lncRNAs to function in cancer development and progression. For instance, MIR22HG has been demonstrated to promote CD8 T-cell infiltration and acts as a tumor suppressor in cancer (22). NKILA lncRNA can promote tumor immune evasion by sensitizing T cells to activation-induced cell death (57). Moreover, pan-adult cancer analysis also revealed that immune-related lncRNAs were likely to be correlated with immune cell infiltrations (20). In addition, we also revealed that immune cell infiltration-related lncRNAs were potentially regulating genes in immune pathways. Identification of these lncRNAs provided a valuable resource for the functional characterization of lncRNA regulation in immunology through further experiments in cell lines or animal models.

Previous studies have revealed the critical roles of the N6-methyladenosine (m6A) modification of lncRNAs in various types of cancers (58, 59). We revealed that the numerous expressions of lncRNAs were associated with DNA methylation, such as PART1, RAMP2-AS1, DLGAP1-AS1, and DLEU1 (**Figure S14**). These lncRNAs have been demonstrated to be associated with brain tumors. For example, PART1 exerts tumor-suppressive functions in glioma *via* sponging miR-190a-3p and inactivation of the PTEN/AKT pathway (60). Knockdown of DLEU1 inhibits glioma progression and promotes temozolomide chemosensitivity by regulating autophagy (61). We next queried the immune-related lncRNAs in m6A-Atlas, which is a comprehensive knowledgebase for unraveling the m6A epitranscriptome (62). We found that several lncRNAs were correlated with m6A modification, such as SSTR5-AS1, MIF-AS1, and CARMN (**Table S5**). Collectively, these findings highlight the critical role of the DNA methylation and m6A modification in regulating lncRNAs, providing a new way to explore RNA epigenetic regulatory patterns in the future.

In conclusion, our study comprehensively analyzes the lncRNA methylation landscape across pediatric pan-CNS tumors and gives first indications of the potential of lncRNA methylation as an adjunct to tumor classification. The repertoire of epigenetically regulated immune-related lncRNAs will facilitate the development of immunotherapeutic targets in pediatric pan-CNS tumors.

## Data Availability Statement

The datasets presented in this study can be found in online repositories. The names of the repository/repositories and accession number(s) can be found in the article/**Supplementary Material**.

## Author Contributions

WX, XL, and KL designed the study. YL, SX, and DX analyzed and interpreted the data. TP, JG, and SX performed the immune analysis. SG and QL designed the figures. YL, XL, and WX wrote and edited manuscript. All authors contributed to the article and approved the submitted version.

## Funding

This work was supported by the Hainan Province Science and Technology Special Fund (Nos. ZDYF2021SHFZ088, ZDYF2021SHFZ051 and ZDYF2020225), Major Science and Technology Program of Hainan Province (Nos. ZDKJ2019010 and hxk200020), the Hainan Provincial Natural Science Foundation of China (Nos. 820MS053 and 820RC637), project supported by Hainan Province Clinical Medical Center (QWYH202175), the National Key R&D Program of China (No. 2018YFC2000100), the Hainan Medical and Health Research Project (No. 2001032034A2004), the National Natural Science Foundation of China (Nos. 32160152, 31970646, 61873075, 32060152, 32070673, and 31871338), the Natural Science Foundation for Distinguished Young Scholars of Heilongjiang Province (No. JQ2019C004), and the Heilongjiang Touyan Innovation Team Program and Innovation Research Fund for Graduate Students (Nos. Hys2020-369, Qhys2021-348, Qhys2021-350, Qhys2021-351, Qhys2021-377, HYYB2021A01, and HYYS2021A31). We are grateful to the tremendous supports from Hainan Excellent Talent Team Project (Child health and early childhood development-The first 1000 days of life).

## Conflict of Interest

The authors declare that the research was conducted in the absence of any commercial or financial relationships that could be construed as a potential conflict of interest.

## Publisher’s Note

All claims expressed in this article are solely those of the authors and do not necessarily represent those of their affiliated organizations, or those of the publisher, the editors and the reviewers. Any product that may be evaluated in this article, or claim that may be made by its manufacturer, is not guaranteed or endorsed by the publisher.
