# Supplementary material for: Pediatric Pan-Central Nervous System Tumor Methylome Analyses Reveal Immune-Related LncRNAs
Source: Front Immunol. 2022 May 4;13:853904. doi: 10.3389/fimmu.2022.853904 (PMC9114481; doi:10.3389/fimmu.2022.853904)
Supplement: Supplementary file 1 [file DataSheet_1.docx]

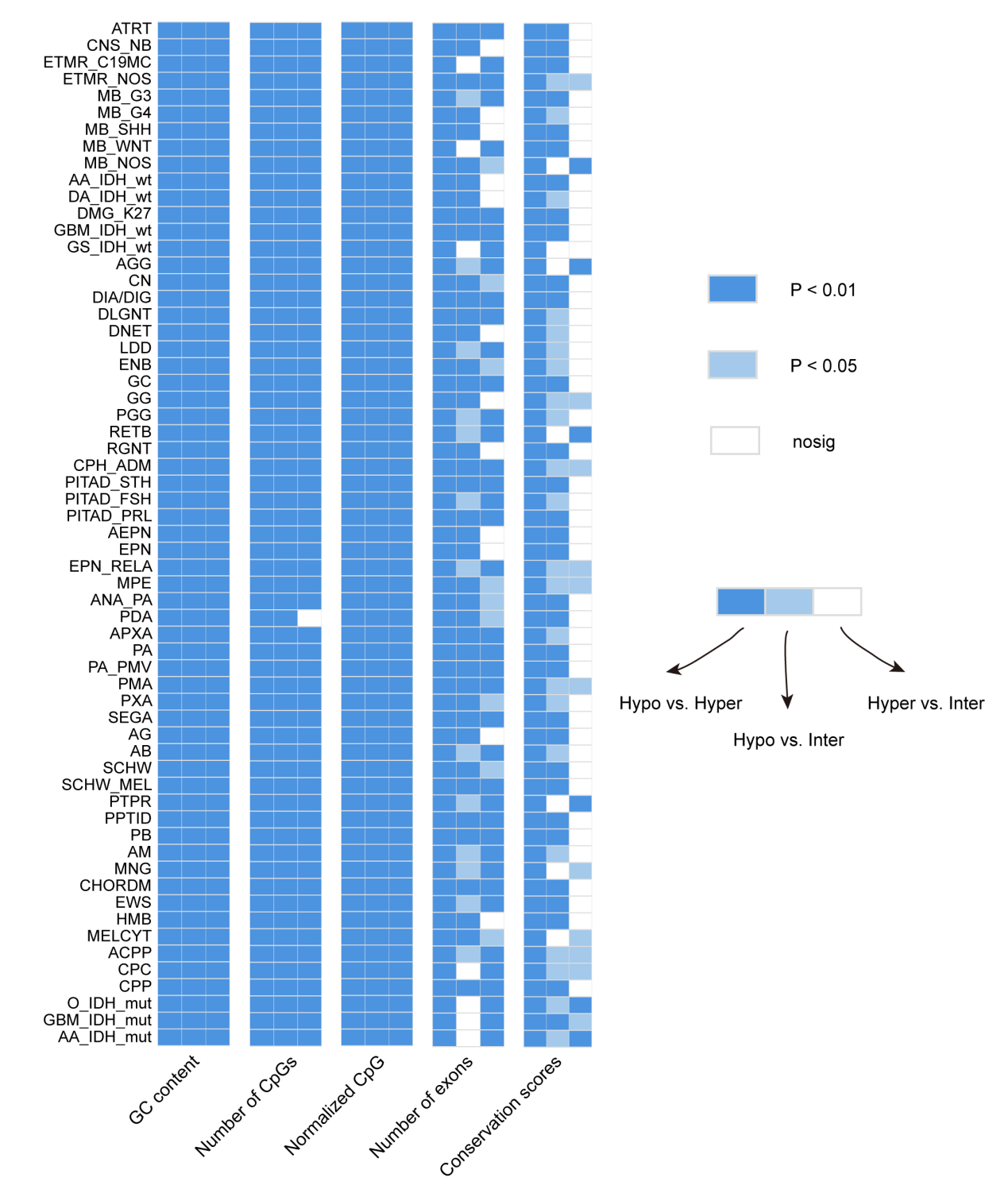


**Figure S1. Comparison of genomic features among lncRNAs groups across 61 pediatric pan-CNS tumors.** The differences in GC contents and normalized CpG fractions were evaluated by Kolmogorov–Smirnov tests. Differences in conservation scores, number of CpGs and exons were evaluated by Wilcoxon’s rank sum tests.


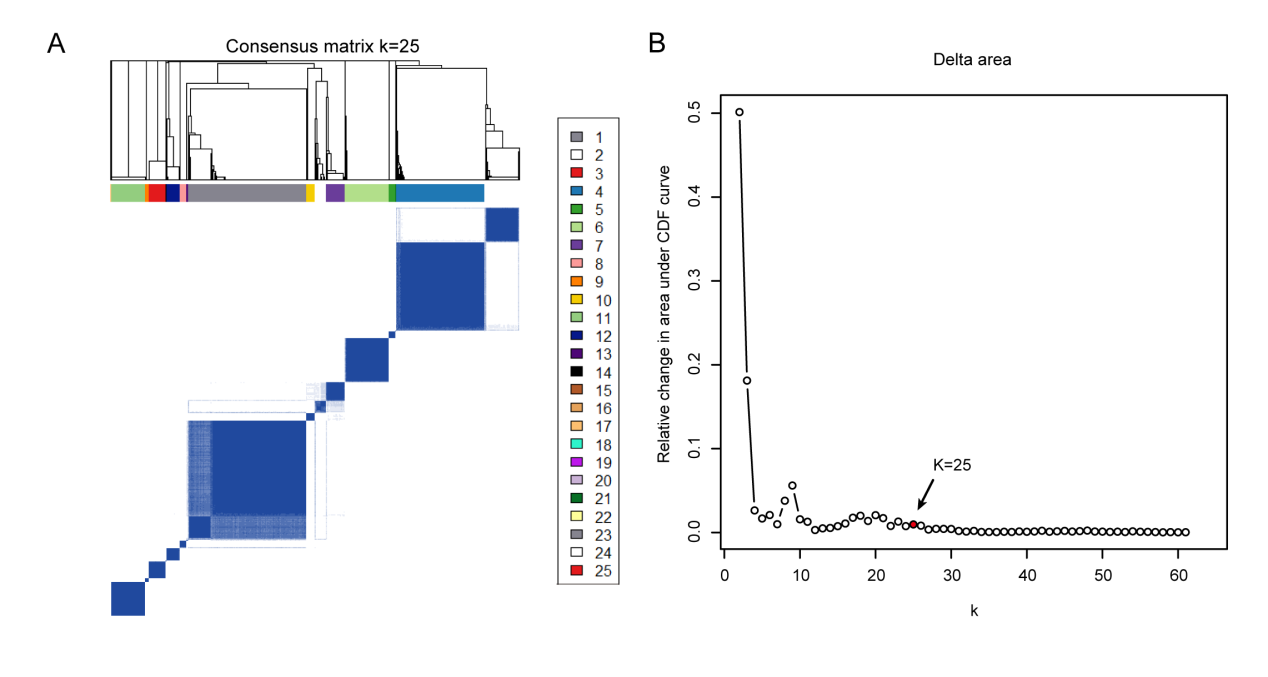


**Figure S2. LncRNA methylation defines stable clusters for pediatric pan-CNS tumors. A,** Consensus clustering matrix of prostate cancer samples for k = 25. **B,** Relative change in area under CDF curve for k = 2 to k=61.


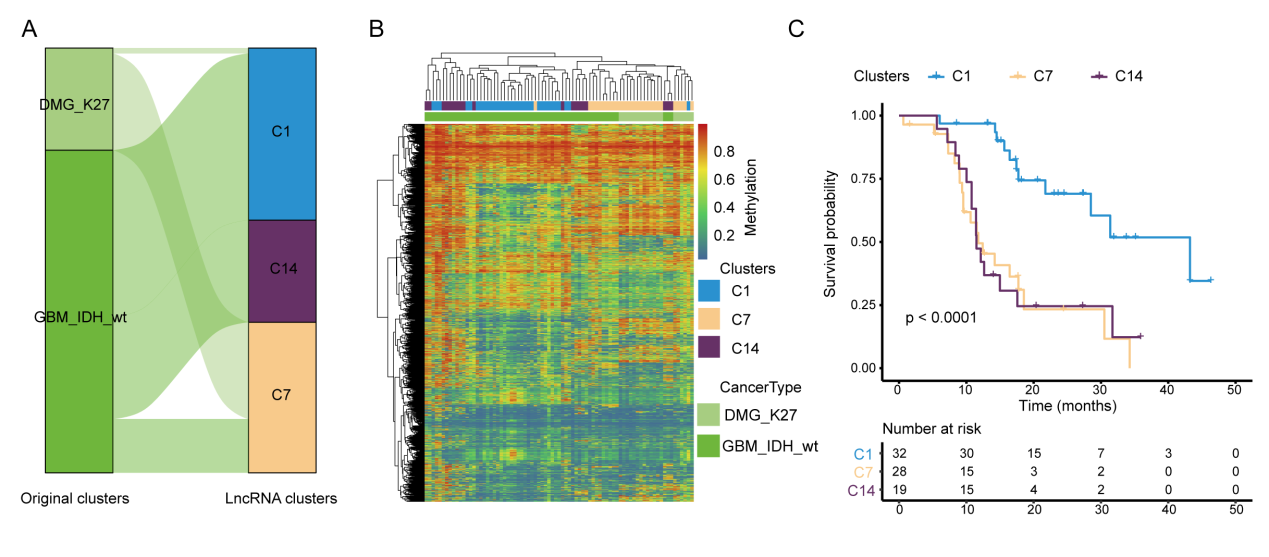


**Figure S3. LncRNA methylation heterogeneity in pediatric giloma tumors.** **A,** Alluvial diagram of lncRNA methylation clusters in groups with different molecular subtypes. **B,** Heat maps showing the methylation of lncRNAs in different clusters. **C,** Kaplan-Meier curves of overall survival for patients with lncRNA methylation clusters.


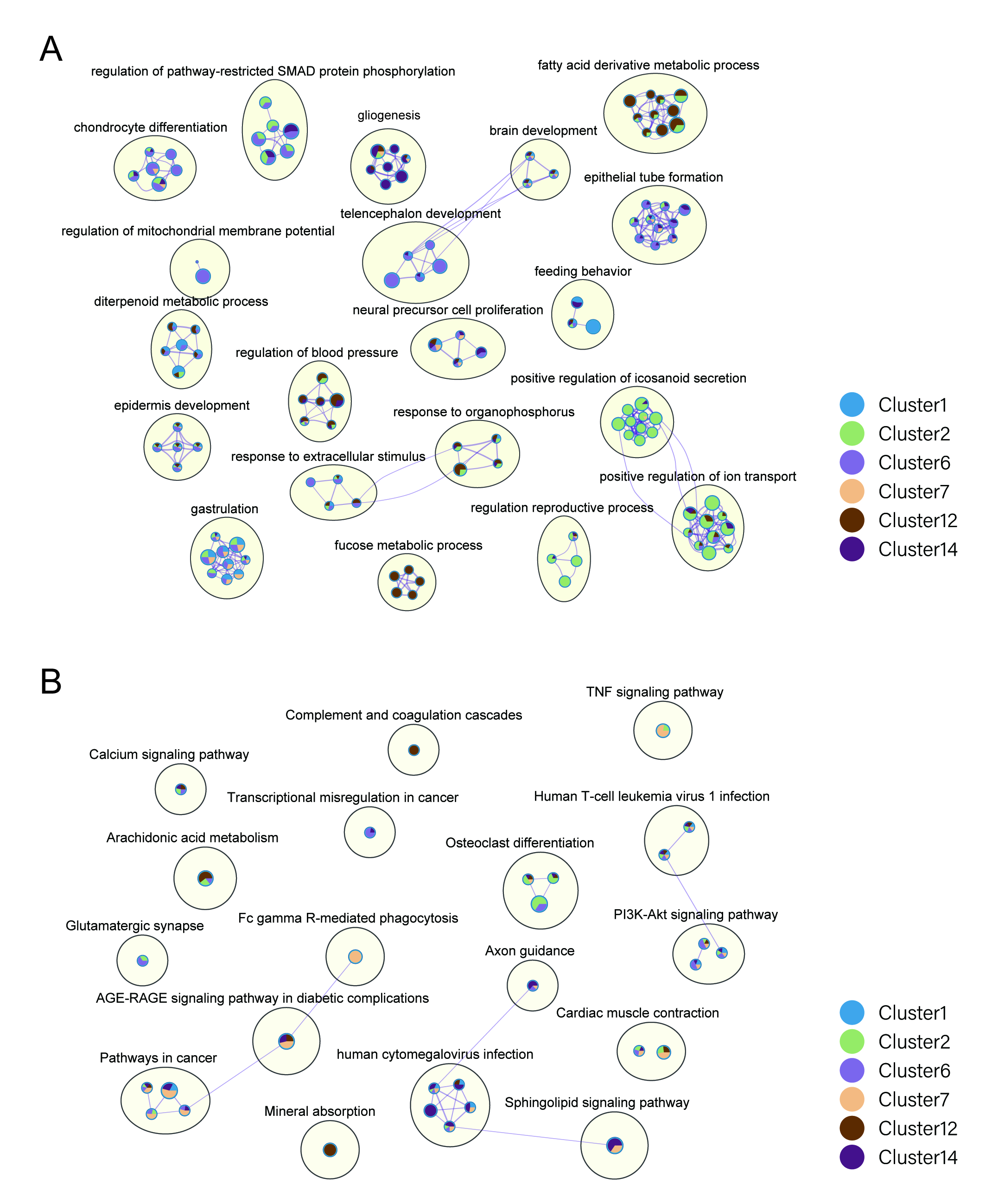


**Figure S4. Functional analysis of cluster-specific QDMRs based on lncRNA methylation. A,** Biological processes enriched by the genes in cluster-specific QDMRs. **B,** KEGG pathways enriched by the genes in cluster-specific QDMRs.


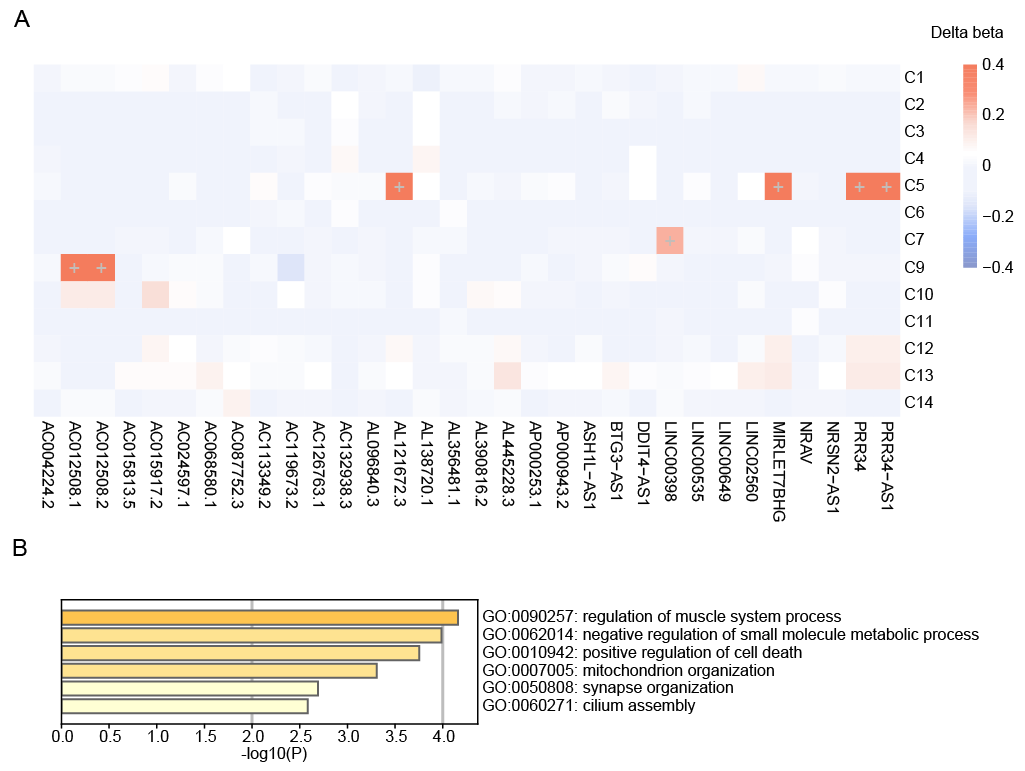


**Fig. S5. Differential methylation of lncRNAs and functions enriched by correlated genes.** A, Heat map showing the difference in DNA methylation. B, Functions enriched by lncRNA correlated genes.


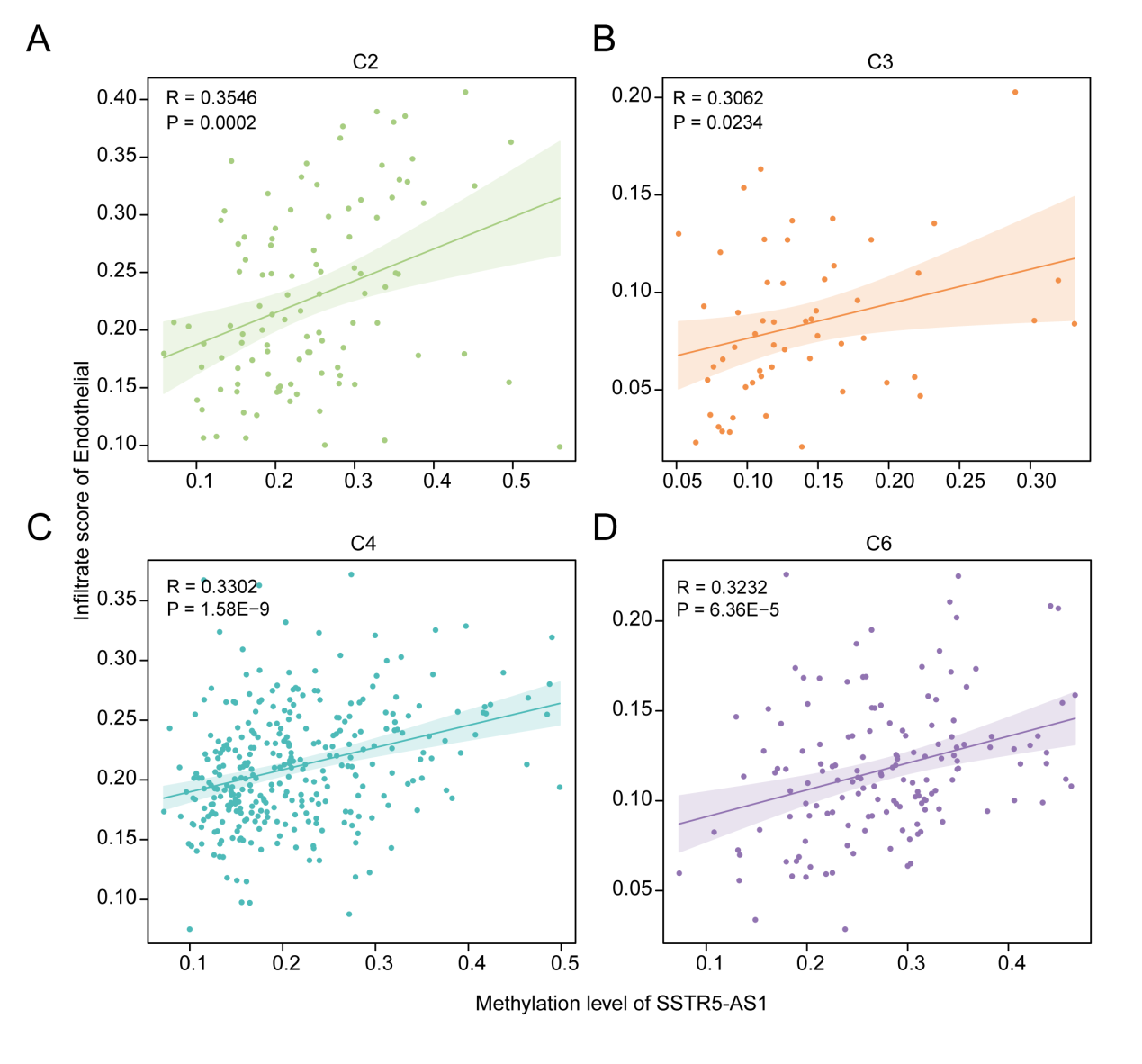


**Figure S6. The association between the methylation of SSTR5-AS1 and the infiltrate score of Endothelial in pediatric medulloblastoma. A** for C2 Cluster, **B** for C3 Cluster, **C** for C4 Cluster and **D** for C6 Cluster.


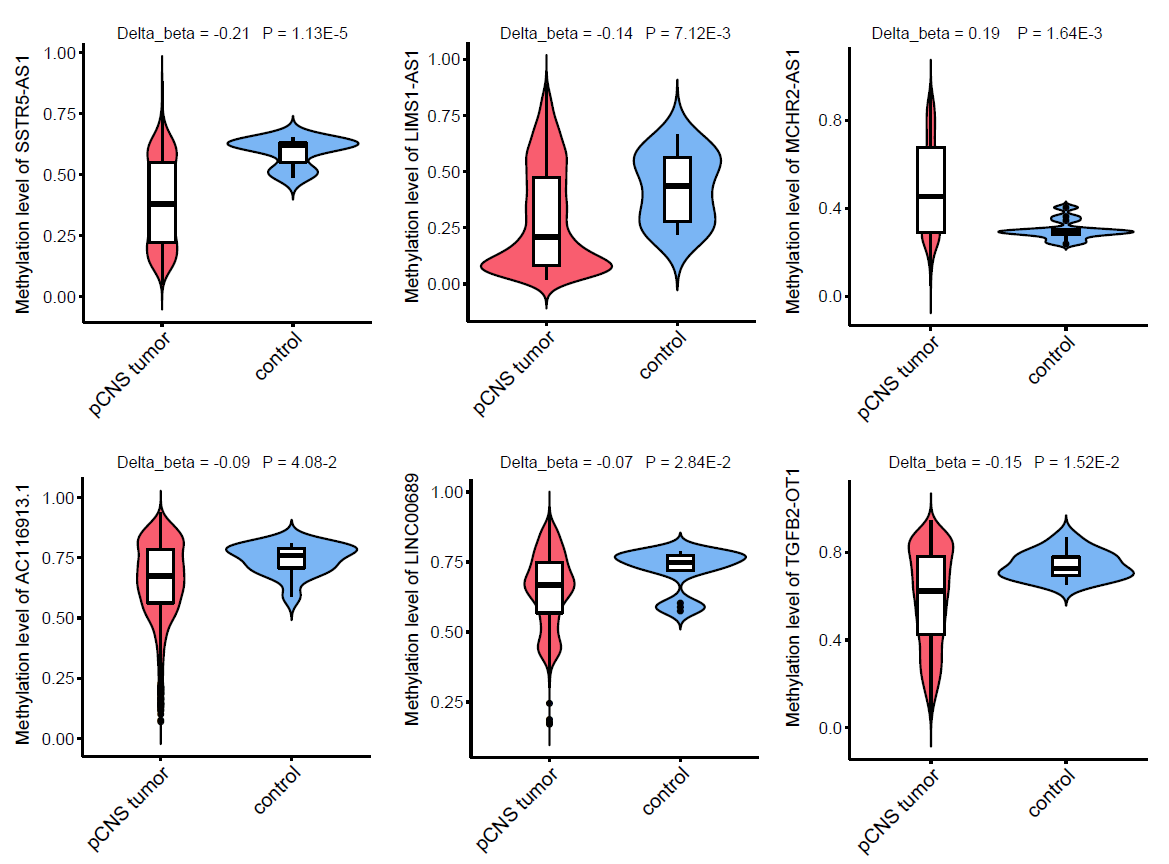


**Figure S7. DNA methylation of representative lncRNAs in tumor and normal controls.**


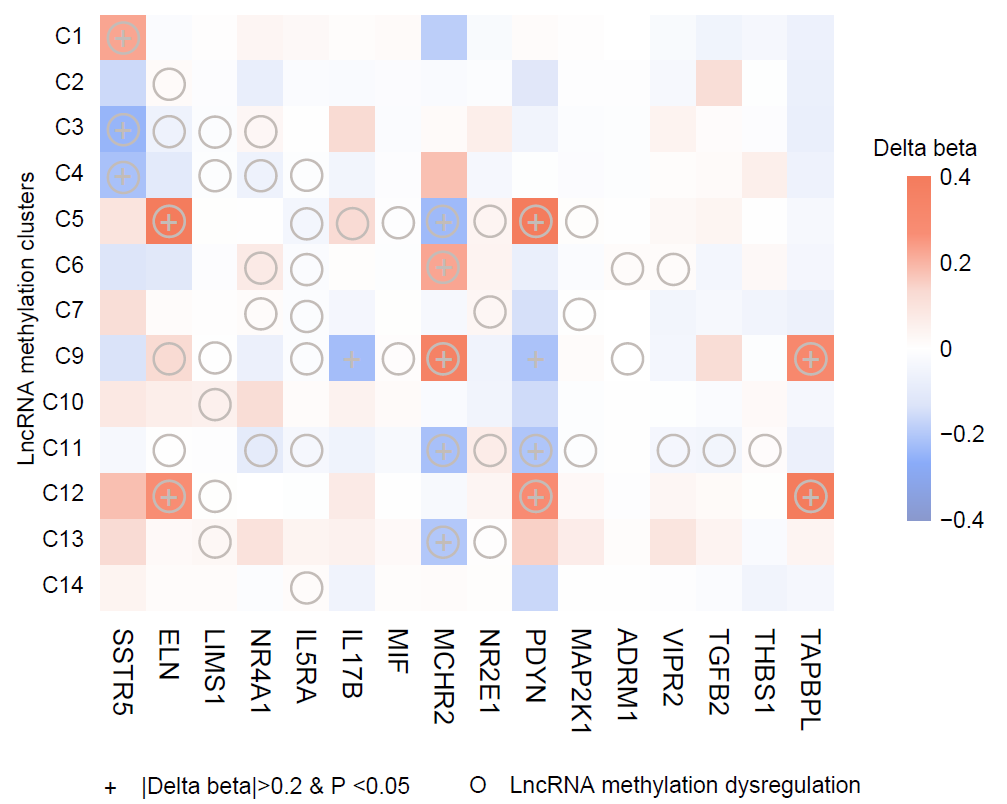


**Fig. S8. DNA methylation differences of genes in immune pathways across different clusters. Circles indicated that lncRNA were dysregulated in corresponding clusters.**


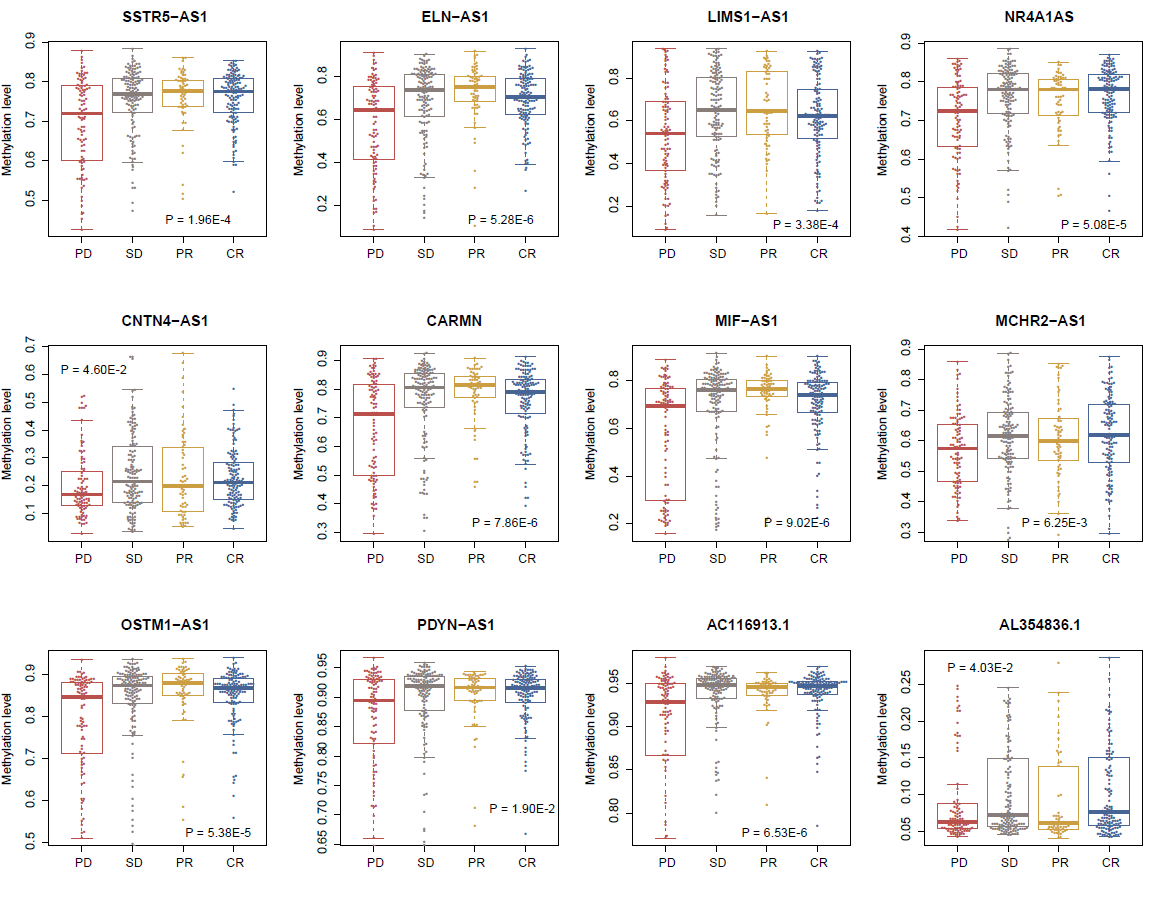


**Figure S9. DNA methylation of representative lncRNAs in tumors with different responses to drug treatments.** PD, progression diseases; SD, stable disease; PR, partial response; CR, complete response.


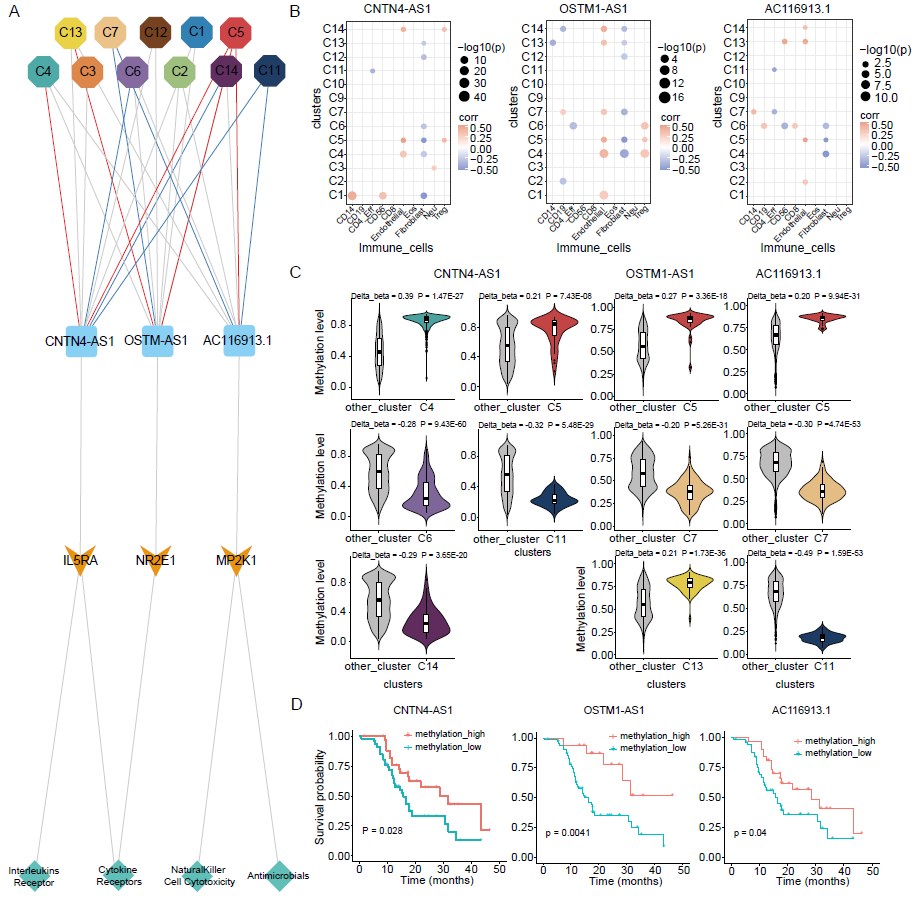


**Fig. S10. Representative examples of lncRNA-gene-pathway in cancer. A,** Hierarchical structure of lncRNA-gene-pathway. **B,** Correlation of lncRNA methylation with immune cell infiltrations. **C,** Boxplots showing the methylation levels of lncRNAs. **D,** Kaplan-Meier curves of overall survival for patients with high or low lncRNA methylation.


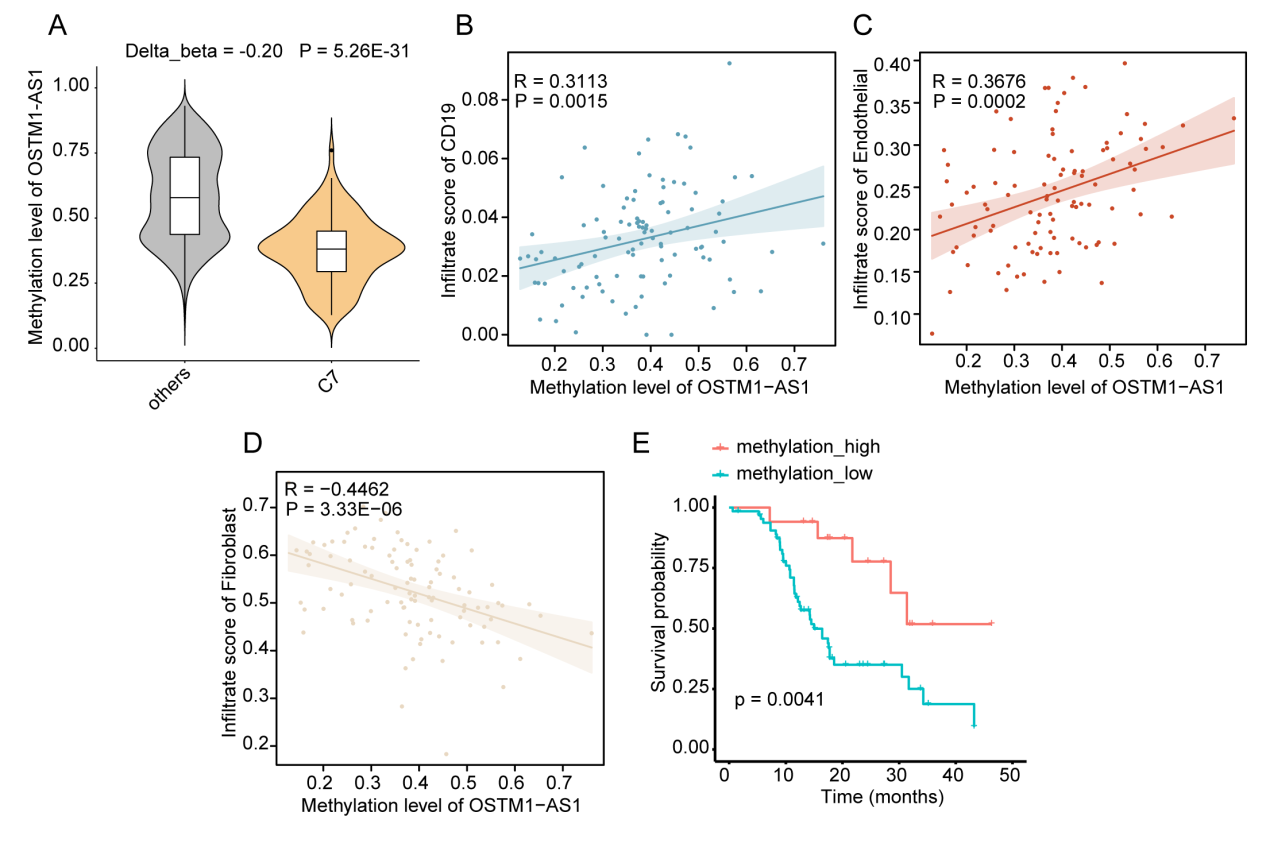


**Figure S11. OSTM1-AS1 methylation involved in immune-related pathways. A,** Violin plots showing the methylation levels of OSTM1-AS1 in C7 and other clusters. **B, C** and **D,** Scatter plots showing the correlation between methylation of OSTM1-AS1 and infiltration of immune cells. **B** for CD19, **C** for endothelial and **D** for fibroblast cells. **E,** Kaplan-Meier curves of overall survival for patients with high or low OSTM1-AS1 methylation.


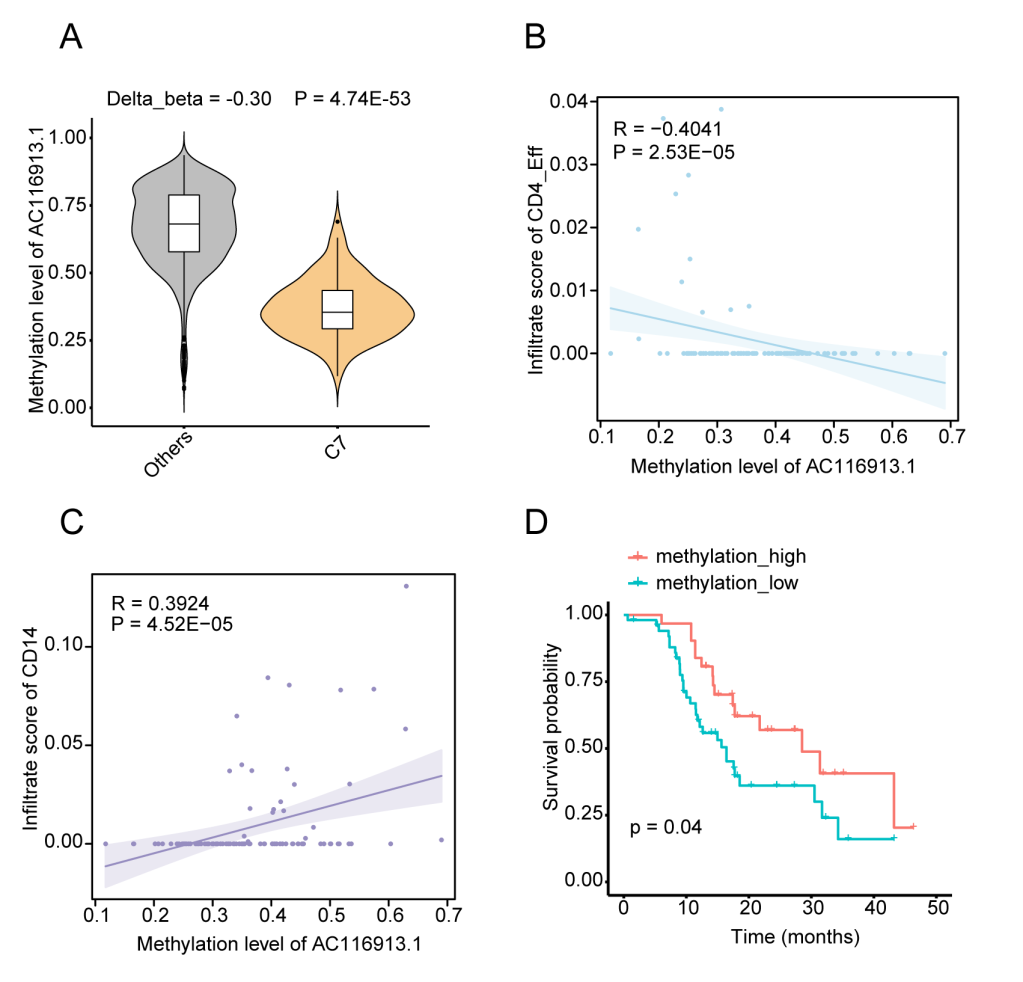


**Figure S12. AC116913.1 methylation involved in immune-related pathways. A,** Violin plots showing the methylation levels of AC116913.1 in C7 and other clusters. **B** and **C,** Scatter plots showing the correlation between methylation of AC116913.1 and infiltration of immune cells. **B** for CD4_Eff and **C** for CD14 cells. **D,** Kaplan-Meier curves of overall survival for patients with high or low AC116913.1 methylation.


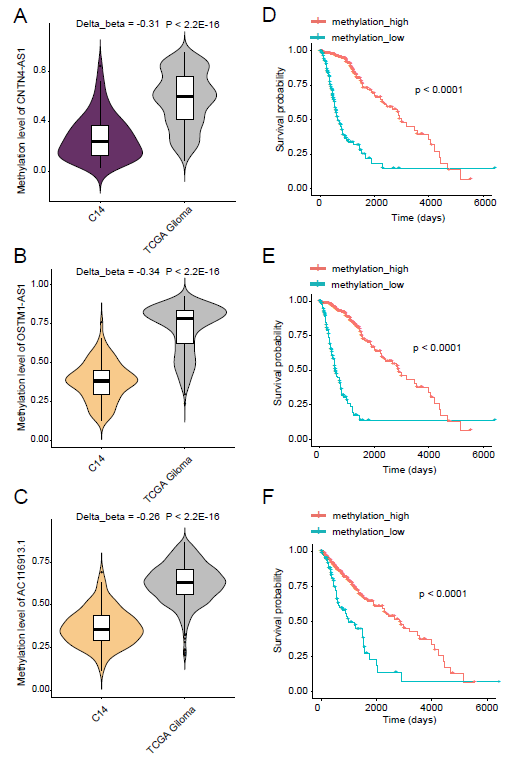


**Fig. S13. DNA methylation levels and association with survival of brain tumors.** A-C, Boxplots showing the DNA methylation levels of lncRNAs. D-F, Kaplan-Meier curves of overall survival for patients with high or low lncRNA methylation.


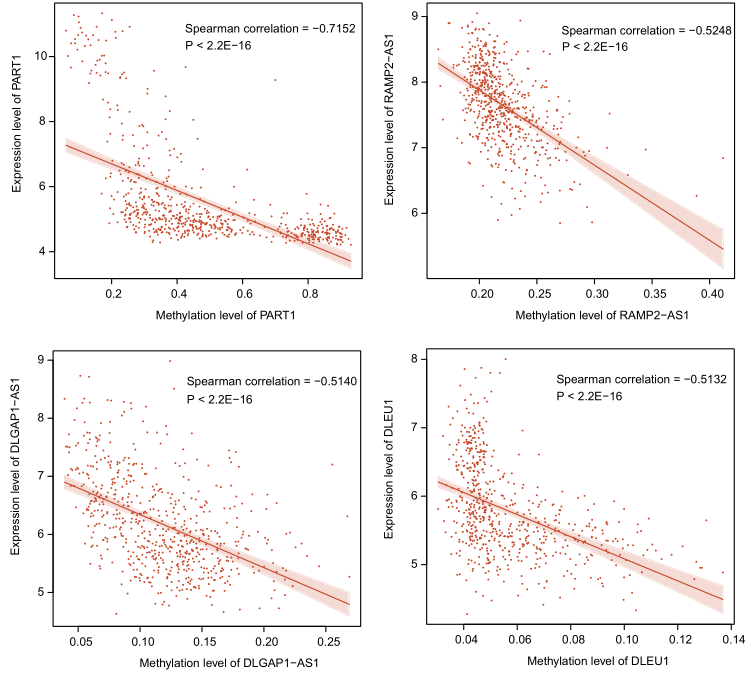


**Fig. S14. Correlation between lncRNA methylation and expression.**
